# Identification of genetic variants predictive of early onset pancreatic cancer through a population science analysis of functional genomic datasets

**DOI:** 10.18632/oncotarget.10924

**Published:** 2016-07-29

**Authors:** Jinyun Chen, Xifeng Wu, Yujing Huang, Wei Chen, Randall E. Brand, Ann M. Killary, Subrata Sen, Marsha L. Frazier

**Affiliations:** ^1^ Department of Epidemiology The University of Texas M. D. Anderson Cancer Center, Houston, Texas, USA; ^2^ Department of Gastroenterology, Hepatology, and Nutrition, The University of Pittsburgh, Pittsburgh, Pennsylvania, USA; ^3^ Department of Translational Molecular Pathology, The University of Texas M. D. Anderson Cancer Center, Houston, Texas, USA; ^4^ Program in Human and Molecular Genetics, The University of Texas Graduate School of Biomedical Sciences, Houston, Texas, USA

**Keywords:** SNP, genetic variant, age at diagnosis, pathway, pancreatic cancer

## Abstract

Biomarkers are critically needed for the early detection of pancreatic cancer (PC) are urgently needed. Our purpose was to identify a panel of genetic variants that, combined, can predict increased risk for early-onset PC and thereby identify individuals who should begin screening at an early age. Previously, we identified genes using a functional genomic approach that were aberrantly expressed in early pathways to PC tumorigenesis. We now report the discovery of single nucleotide polymorphisms (SNPs) in these genes associated with early age at diagnosis of PC using a two-phase study design. *In silico* and bioinformatics tools were used to examine functional relevance of the identified SNPs. Eight SNPs were consistently associated with age at diagnosis in the discovery phase, validation phase and pooled analysis. Further analysis of the joint effects of these 8 SNPs showed that, compared to participants carrying none of these unfavorable genotypes (median age at PC diagnosis 70 years), those carrying 1–2, 3–4, or 5 or more unfavorable genotypes had median ages at diagnosis of 64, 63, and 62 years, respectively (*P* = 3.0E–04). A gene-dosage effect was observed, with age at diagnosis inversely related to number of unfavorable genotypes (*P*_trend_ = 1.0E–04). Using bioinformatics tools, we found that all of the 8 SNPs were predicted to play functional roles in the disruption of transcription factor and/or enhancer binding sites and most of them were expression quantitative trait loci (eQTL) of the target genes. The panel of genetic markers identified may serve as susceptibility markers for earlier PC diagnosis.

## INTRODUCTION

Pancreatic cancer (PC) is the fourth leading cause of cancer-related death in the United States. An estimated 53,070 new cases and 41,780 deaths due to this disease are expected in the United States in 2016 [1]. Because of the asymptomatic onset of pancreatic cancer and absence of reliable biomarkers for early detection, most patients already have late-stage or metastatic disease at the time of diagnosis, resulting in an overall 5-year survival rate of only 7.2% [2]. Although for most cancers there have been notable improvements in survival over the past 3 decades, PC has shown little improvement [1]. Biomarkers for the early detection of PC are urgently needed. A better understanding of the molecular mechanisms underlying PC tumorigenesis would help in development of early detection strategies as well as more meaningful diagnostic and prognostic markers.

As a part of the National Cancer Institute Early Detection Research Network, our goal is to assemble a panel of candidate blood-based biomarkers for early detection of PC. Our premise is that uncovering the earliest genetic pathways aberrant in PC could reveal a clinically useful panel of biomarkers. We have focused on intervals of recurrent cytogenetic loss and gain associated with deletion/loss of function of tumor suppressor loci and overexpression/gain of function of oncogenes. Our objective has been to identify recurrent alterations that fall within an early cytogenetic pathway to PC tumorigenesis. Published studies suggest that multiple tumor types share a common 3p12 pathway to tumorigenesis and that regions of loss and amplification of chromosome 20q occur early in tumorigenic transformation and may initiate cancer [3–8]. We have chosen to concentrate on these pathways in our search for potential biomarkers. Significantly, early losses of chromosome 3p or 1p and amplification of chromosome 20q have been reported in smoking-related cancers [9, 10].

Members of the 3p12 pathway and genes related to the chromosome 20q interval are important in pancreatic tumorigenesis [3, 7, 8], and their combined influence is believed to contribute to the process of pancreatic tumorigenesis. It is therefore reasonable to hypothesize that combinations of genetic variants in these genes may help in driving the process of tumorigenesis. Our previous studies indicate that single nucleotide polymorphisms (SNPs) in several different genes, including *SEL1L*, *Aurora-A*, *p16*, *p21*, and *p27*, can modify the age at diagnosis of PC [12–14]. We hypothesized that variants of genes involved in the 3p12 and 20q pathways may work together to modify age at diagnosis of PC. We previously utilized functional genomic pathways approaches to identify chromosome 3p12 pathway and chromosome 20q pathway genes as candidate early detection biomarkers, which could discriminate PC from healthy controls (11 and unpublished). To test our hypothesis, we first selected tagging SNPs for these genes based on observed linkage disequilibrium (LD) through construction of metric LD maps. Secondly, we used a two-phase study design to identify SNPs in these genes associated with early age at diagnosis of PC in total 1729 PC patients. Thirdly, we utilized *In silico* and bioinformatics tools for examining functional relevance of SNPs we identified.

**Figure 1:**
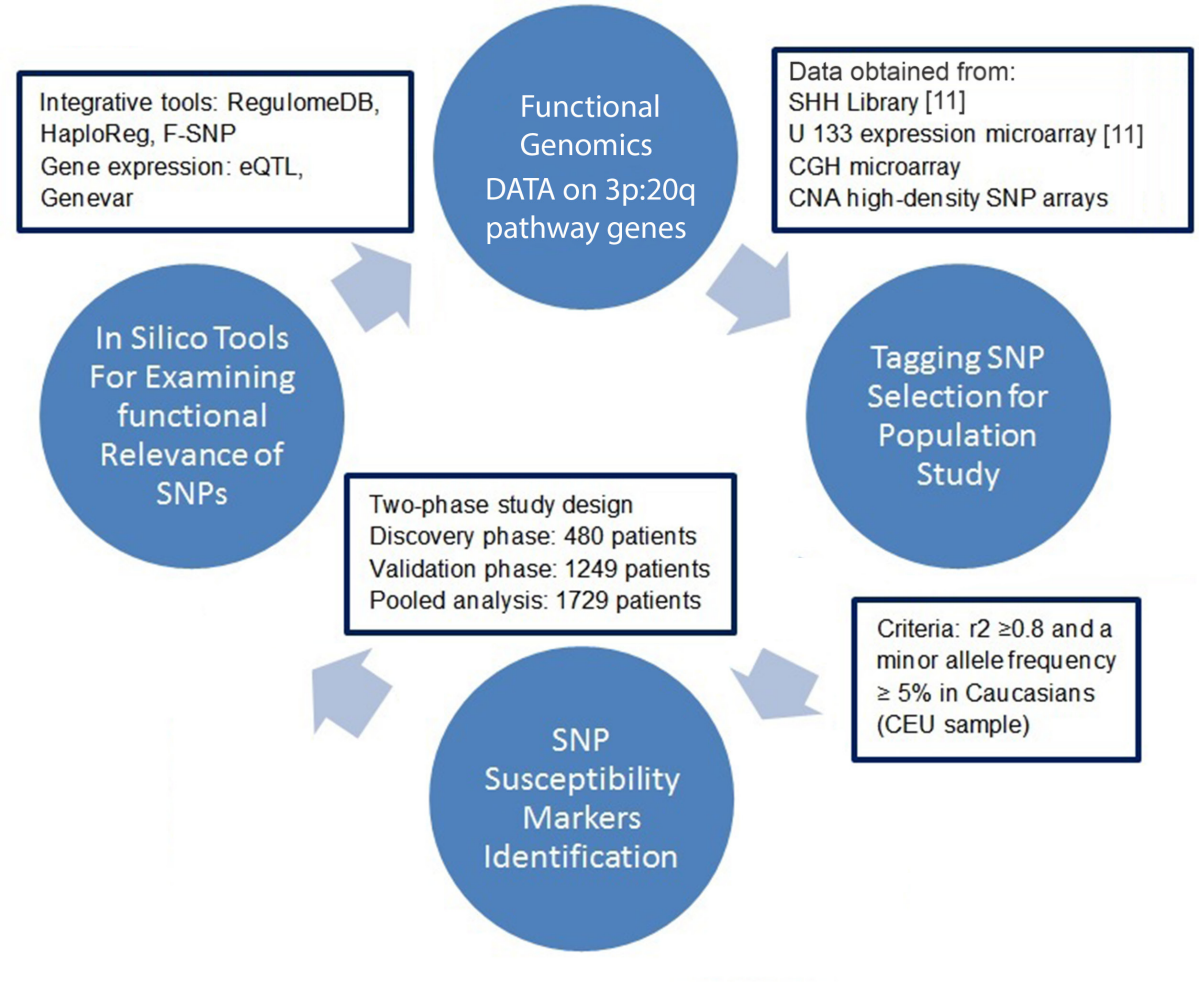
A schematic flow diagram of functional genomic data and population epidemiology method to identify genetic variants as susceptibility markers for earlier pancreatic cancer diagnosis CGH: comparative genomic hybridization; CNA: copy number alterations; SSH: suppression subtractive hybridization; eQTL: expression quantitative trait loci.

Our study integrated data from a systems biology approach and population science methods to discover genetic variants as susceptibility markers for earlier pancreatic cancer diagnosis. Then, we functionally analyzed the susceptibility loci using *in silico* and bioinformatics tools. The study design flow diagram is shown in Figure 1. We identified a panel of genetic risk factors, i.e., SNPs, and other relevant covariates that, in combination, can predict risk for early age onset of PC and thereby identify individuals who might benefit from screening at an early age.

## RESULTS

### Participant characteristics

The demographic characteristics and PC risk factor status of participants are shown in Table 1. We restricted the analysis to self-reported non-Hispanic white participants to minimize confounding by ethnicity. The discovery (phase 1) analysis and the validation (phase 2) analysis included 480 and 1249 randomly selected patients, respectively. There was no overlap of subjects in the discovery phase and the validation phase. The mean ages at diagnosis in phase 1 and phase 2 were 61.6 and 64.2 years, respectively. Age at diagnosis was 50 years or younger for more than 10% of participants in both phases 1 and 2. The proportion of smokers was higher than the proportion of non-smokers in both phases (phase 1, 59.4%; phase 2, 54.7%), as was the proportion of alcohol users (phase 1, 61.9%; phase 2, 57.9%) (Table 1).

**Table 1:** Demographic characteristics and risk factors associated with pancreatic cancer cases

Variable	Discovery Phase	Validation Phase	Pooled Analysis
	No. of Participants (%)	No. of Participants (%)	No. of Participants (%)
	*N* = 480	*N* = 1249	*N* = 1729
**Mean age at diagnosis, years**			
± SD	61.6 ± 10.8	64.2 ± 11.1	63.5 ± 11.1
**Median age at diagnosis, years**			
(range)	62 (21–87)	64, 24–91	64, 21–91
**Age groups at diagnosis**			
≤ 50 years	67 (13.9)	128 (10.2)	195 (11.3)
51 ~ 60 years	129 (26.9)	301 (24.1)	430 (24.9)
60 ~ 70 years	179 (37.3)	427 (34.2)	606 (35.0)
> 70 years	105 (21.9)	393 (31.5)	498 (28.8)
**Sex**			
Female	171 (35.6)	541 (43.3)	712(41.2)
Male	309 (64.4)	708 (56.7)	1017(58.8)
**Diabetes**			
No	339 (70.6)	862 (69.0)	1201 (69.5)
Yes	141 (29.4)	387 (31.0)	528 (30.5)
**Smoking status**			
Never	195 (40.6)	566 (45.3)	761 (44.0)
Former	213 (44.4)	532 (42.6)	745 (43.1)
Current	72 (15.0)	151 (12.1)	223 (12.9)
**Alcohol status**			
Never	183 (38.1)	526 (42.1)	709 (41.0)
Former	135 (28.1)	293 (23.5)	428 (24.8)
Current	162 (33.8)	430 (34.4)	592 (34.2)

### SNPs and age at diagnosis

In phase 1, among 1240 tested SNPs, a total of 149 SNPs were significantly associated with age at diagnosis of PC (*P* < 0.05) by Cox regression analysis adjusted for sex, smoking and alcohol use, and presence of diabetes (Supplementary Table 1). In phase 2, 11 of these 149 SNPs were replicated, showing significant association with age at diagnosis (*P* < 0.05). After the Benjamini-Hochberg false discovery rate correction, SNP rs61992671 in miR-412 remained strongly associated with age at diagnosis of PC, with a *P*-value of 7.19 × 10^−5^, hazard ratio (HR) = 1.28 (95% confidence interval [CI] = 1.14–1.45), corrected *P* = 0.011 (Supplementary Table 2). In a pooled analysis for phases 1 and 2 (*N* = 1729), 25 SNPs showed significant association with age at diagnosis (*P* < 0.05). The association remained significant for 8 SNPs (rs61992671 in miR-412, rs2766669 in *ZNF217*, rs6128327 in *RAB22A*, rs2282544 in *SMAD4*, rs1076064 in *PPARGC1B*, rs7799635 in *KDELR2*, rs4940086 in *SMAD2*, and rs3217992 in *CDKN2B*) after multiple testing correction (threshold with false discovery rate of 10%), with *P*-values of 2.09 × 10^−6^, 8.11 × 10^−4^, 1.07 × 10^−3^, 2.66 × 10^−3^, 2.80 × 10^−3^, 3.30 × 10^−3^, 3.45 × 10^−3^, and 4.93 × 10^−3^, respectively (Supplementary Table 3). Altogether, we found 8 SNPs (rs61992671 in miR-412, rs2766669 in *ZNF217*, rs6128327 in *RAB22A*, rs7799635 in *KDELR2*, rs4940086 in *SMAD2*, rs3217992 in *CDKN2B*, rs12803915 in miR-612, and rs1559849 in *SERAC1*) that were consistently associated with age at diagnosis in the discovery phase, validation phase, and pooled analysis, with *P*-values < 0.05 (Table 2).

**Table 2:** SNPs significantly associated with age at diagnosis of pancreatic cancer in the discovery phase, validation phase, and pooled analysis

Gene	SNP ID	Genetic Model	Discovery Phase		Validation Phase		Pooled Analysis	
			Adjusted HR^a^(95% CI)	*P*	Adjusted HR^a^(95% CI)	*P*	Adjusted HR^a^(95% CI)	*P*
miR-412	rs61992671	Dominant	1.29 (1.05–1.58)	0.015	1.28 (1.14–1.45)	7.19E–05	1.29 (1.16–1.43)	2.09E–06
*ZNF217*	rs2766669	Dominant	1.34 (1.10–1.64)	0.005	1.16 (1.02–1.31)	0.021	1.20 (1.08–1.33)	8.11E–04
*RAB22A*	rs6128327	Additive	1.12 (1.05–1.20)	0.008	1.10 (1.01–1.22)	0.023	1.12 (1.05–1.20)	0.001
*KDELR2*	rs7799635	Additive	0.87 (0.75–0.94)	0.042	0.89 (0.81–0.98)	0.013	0.89 (0.82-0.96)	0.003
*SMAD2*	rs4940086	Additive	1.22 (1.01–1.47)	0.040	1.10 (1.00–1.20)	0.040	1.11 (1.04–1.20)	0.003
*CDKN2B*	rs3217992	Recessive	0.78 (0.61–0.99)	0.048	0.84 (0.72–0.98)	0.028	0.83 (0.73–.95)	0.005
miR-612	rs12803915	Additive	1.69 (1.03–2.76)	0.039	1.11 (1.01–1.22)	0.038	1.11 (1.02–1.21)	0.012
*SERAC1*	rs1559849	Recessive	1.25 (1.01–1.54)	0.038	1.85 (1.26–2.72)	0.001	1.47 (1.06–2.04)	0.021

a Adjusted by sex, smoking status, alcohol status, diabetes status, and study phase (for pooled analysis)

SNP, single nucleotide polymorphism; HR, hazard ratio; CI, confidence interval.

To assess the cumulative effects of the unfavorable genotypes on age at diagnosis, we performed a joint analysis of the 8 SNPs that were consistently associated with age at diagnosis in all the analyses. In the pooled analysis (*N* = 1729), we found that, compared with participants carrying no unfavorable genotype (of any of the 8 SNPs), participants carrying 1–2, 3–4, or 5 or more unfavorable genotypes exhibited an 1.63-fold (95% CI = 1.10–2.42, *P* = 0.016), 1.88-fold (95% CI = 1.27–2.78; *P* = 0.002) or 2.11-fold (95% CI = 1.4–3.18, *P* = 3.0 × 10^−4^) increased risk of early PC onset, respectively. The median age at diagnosis differed significantly between the 4 groups: This was 70 years for participants with no unfavorable genotypes, 64 years for those with 1–2 unfavorable genotypes, 63 years for those with 3–4 unfavorable genotypes, and 62 years for those with 5 or more unfavorable genotypes, with a significant dose-response trend (*P* for trend = 1.0 × 10^−4^) (Table 3). The age at diagnosis between participants carrying no unfavorable genotypes and those carrying 5 or more unfavorable genotypes differed by 8 years.

**Table 3:** Cumulative analysis of unfavorable genotypes

No. of Unfavorable Genotypes	No. of Participants	Median Age at Diagnosis, years	Adjusted HR^a^ (95% CI)	*P*
0	26	70	1.00 (reference)	
1–2	528	64	1.63 (1.10–2.42)	0.016
3–4	943	63	1.88 (1.27–2.78)	0.002
≥ 5	227	62	2.11 (1.40–3.18)	3.0E–04
Trend test				1.0E–04

a Adjusted by sex, smoking status, alcohol status, diabetes status, and study phase; HR, hazard ratio.

### SNP function and eQTL analysis

ENCODE data and the F-SNP, HaploReg, and RegulomeDB tools indicate that all of the 8 identified SNPs (rs61992671, rs2766669, rs6128327, rs7799635, rs4940086, rs3217992, rs12803915, and rs1559849) have potential for disruption of transcription factor and/or enhancer binding sites. rs4940086 (*SMAD2)* and rs12803915 (miR-612) are located in potential enhancer regions, based on histone marks, in normal breast, lymphocyte, or leukemia cells. In addition, rs12803915 (miR-612) is located in a “hot spot” of DNase I hypersensitivity sites in 9 cell types and is predicted to affect binding of proteins, including POLR2A, REST, TFAP2C, and ZBTB7A. rs3217992 (*CDKN2B)* and rs7799635 (*KDELR2)* are also located in DNase I hypersensitivity sites. Furthermore, rs7799635 (*KDELR2*) is predicted to affect binding and was linked to expression of the *CTCF* gene target as well as the *cis*-eQTL of *KDELR2*. rs61992671 (miR-412*)* is predicted to be located in a conserved transcription factor binding site and to be an exonic splicing enhancer.

**Figure 2:**
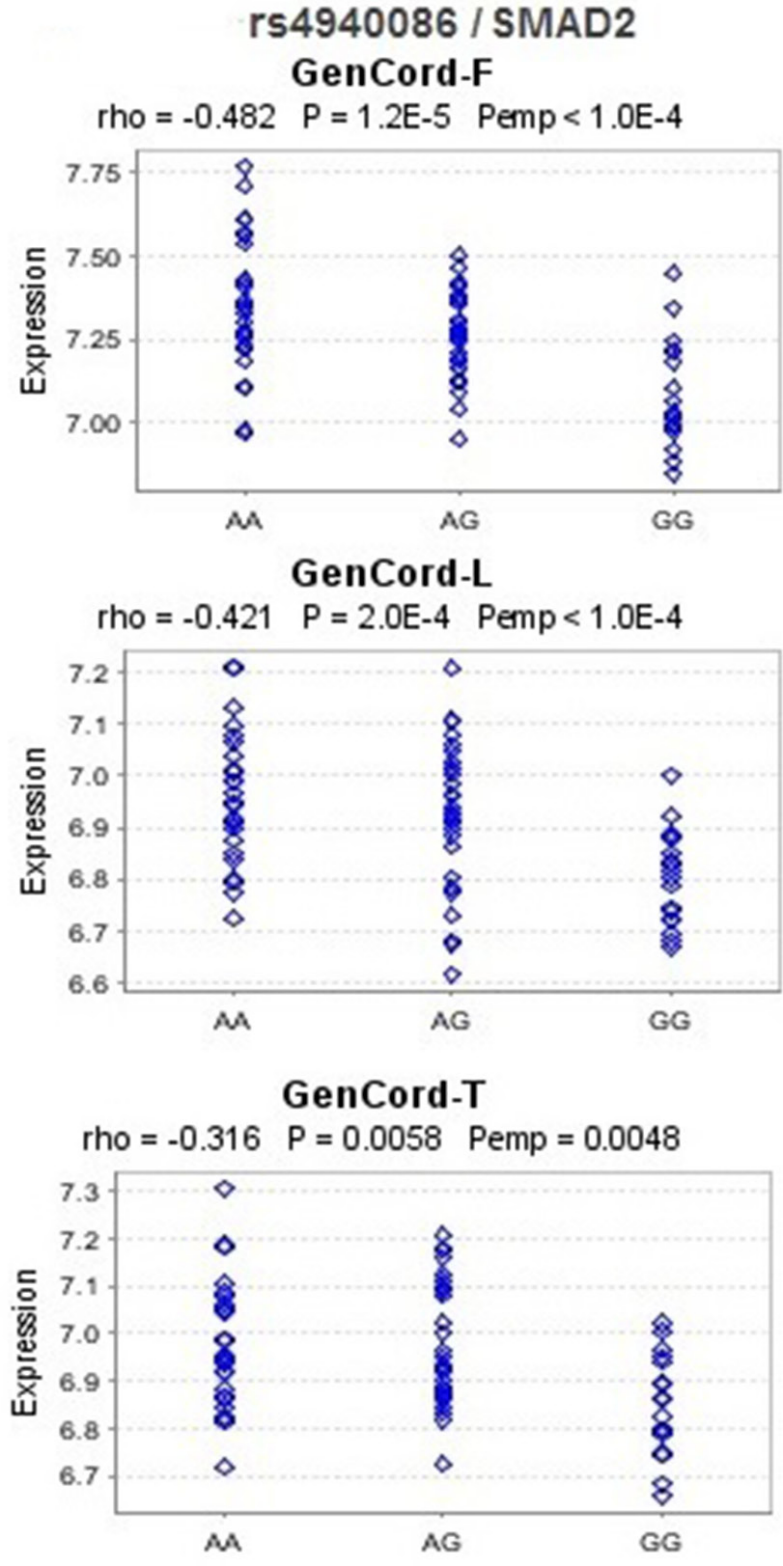
eQTL analysis for rs4940086 and *SMAD2* in the Geneva GenCord study F, L, and T represent fibroblast, lymphoblastoid cell line and T-cell, respectively. Rho: Spearman correlation coefficient; *P*: *P*-values of Spearman correlation test; *P*emp: empirical *P*-values calculated from 10,000 permutations. These figures were downloaded from Genevar.

**Figure 3:**
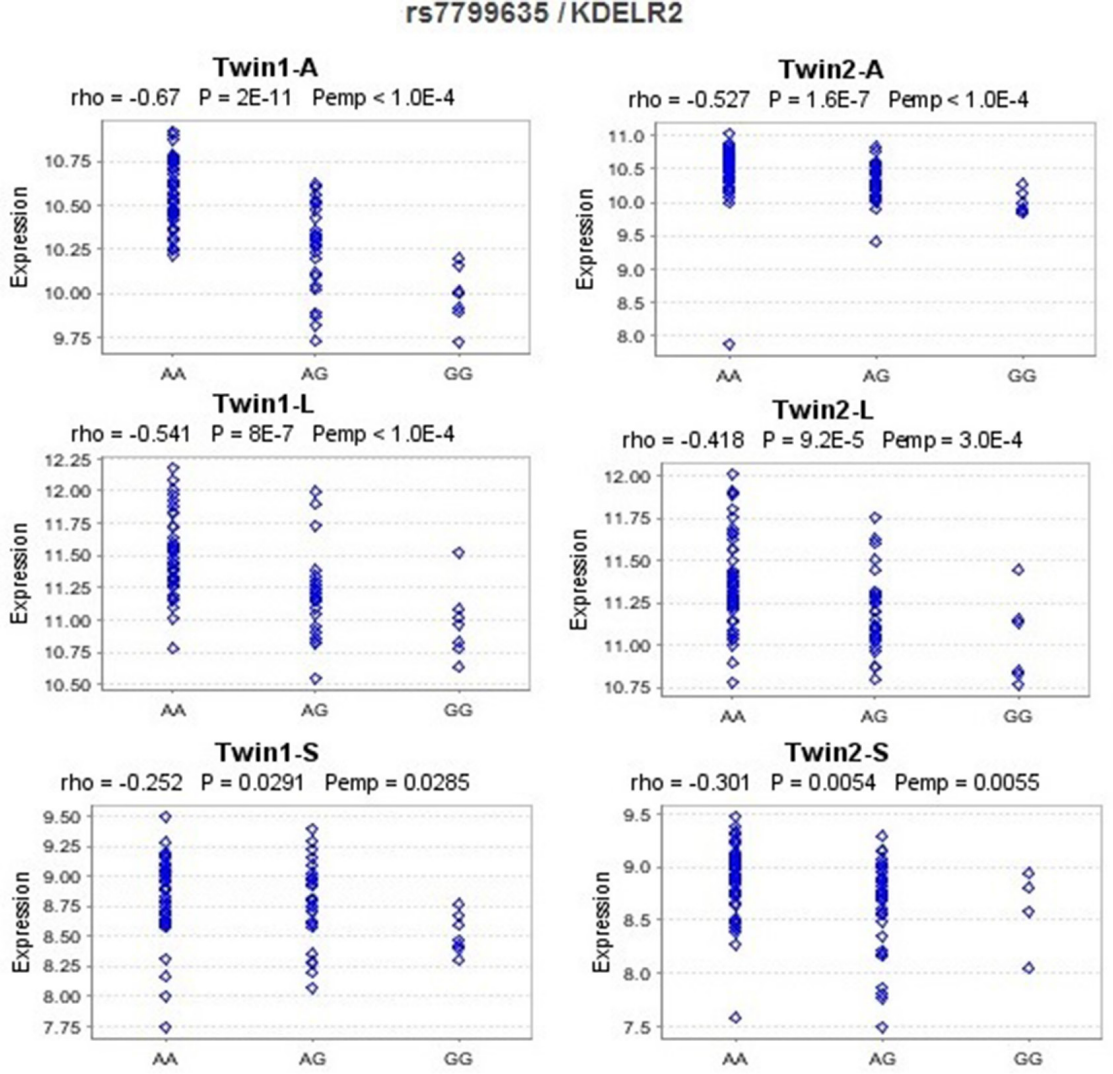
eQTL analysis for rs7799635 and *KDELR2* in the MuTHER pilot study A, L, and S are short for adipose tissue, lymphoblastoid cell line, and skin tissue, respectively, from MuTHER healthy female twins. Rho: Spearman correlation coefficient; *P*: *P*-values of Spearman correlation test; *P*emp: empirical *P*-values calculated from 10,000 permutations. The figures were downloaded from Genevar.

In *cis*-eQTL analysis based on the public dataset Genevar, we found that rs4940086 AA genotype was associated with significantly higher expression level of *SMAD2* in 3 cell types (fibroblasts [*P* = 1.2 × 10^−5^], a lymphoblastoid cell line [*P* = 2.0 × 10^−4^], and T cells [*P* = 0.0058]) derived from umbilical cords of 75 Geneva GenCord individuals [15] than the AG or GG genotypes. The correlations remained significant in 10,000 permutation tests for the 3 cell types (Figure 2). rs7799635 was also significantly associated with the expression level of *KDELR2* in 3 tissue types (adipose, lymphoblastoid cell line, and skin) derived from a subset of ~160 MuTHER healthy female twins [16] (Figure 3). In addition, rs1559849, rs6128327, and rs3217992 were significantly associated with the expression levels of *SERAC1*, *RAB22A*, and *IFNA1* in lymphoblastoid cell lines from 726 HapMap3 [17] (Supplementary Figure 1).

## DISCUSSION

Currently, there is no clinically useful biomarker for earlier diagnosis of PC in the general population. We hypothesized that focusing on aberrantly expressed genes related to early cytogenetic pathways to PC tumorigenesis might be a feasible approach to discover early detection biomarkers. In this study, we utilized functional genomic data which had previously described [11] to discover targeted pathway-based genes and used a two-phase population study design to systematically assess the associations of a large panel of SNPs in the genes that interact in the two targeted pathways with age at diagnosis of PC. Our two-phase study design ultimately identified 8 SNPs consistently associated with age at diagnosis in the discovery phase, validation phase, and pooled analysis. Analysis of the joint effects of these SNPs revealed that participants with more unfavorable genotypes carried higher risk of developing PC at a younger age. The 8-year difference in age at diagnosis between subjects carrying no unfavorable genotypes and those carrying 5 or more unfavorable genotypes suggests that these genetic variants may jointly contribute to an earlier age onset of PC in non-Hispanic white patients. Using bioinformatics tools, we found all of the 8 SNPs were located in DNA sequences with potential functional roles in disruption of transcription factor and/or enhancer binding sites and most of them were eQTL of the targeted genes.

Of the 8 SNPs consistently associated with age at diagnosis, the most significant one is rs61992671, which is found in precursor miR-412. We used miRNA prediction programs (microRNA.org, Targetscan, and Diana-microT v3.0) to predict that miR-412 may target the conserved 3′-untranslated region (UTR) of *Sel-1-like* (*SEL1L*). *SEL1L* is a putative tumor suppressor gene that is downregulated in a significant proportion of human pancreatic ductal adenocarcinomas (PDAC). Our previous studies showed that *SEL1L* was downregulated by aberrantly upregulated hsa-mir-155 in human PDAC [18], and a SNP in *SEL1L* gene plays a role in modifying age at diagnosis of PDAC in white nonsmokers and may serve as a prognostic marker in PDAC patients [12]. *SEL1L* has been reported to play a role in cell transformation and tumor progression in human pancreatic, breast, non–small cell lung, esophageal, and prostate cancers [19–24]. Many human miRNA genes are located in fragile sites or areas of the genome that are frequently associated with cancer. SNPs in precursor miRNA genes may potentially affect the processing of miRNAs and may thus significantly affect risk of cancer [25, 26]. Recently, miR-412 was observed to be upregulated by more than 3-fold in squamous cell lung carcinoma tissues compared with normal tissues [27]. rs61992671 (miR-412*)* was predicted to be located in a conserved transcription factor binding site that may be an exonic splicing enhancer.

The other significant miRNA SNP identified by our study is rs12803915, which is located in precursor miR-612. TargetScan predicts that miR-612 may target the conserved 3′-UTR of (*DEAR1*, ductal epithelium–associated RING chromosome 1 (annotated as TRIM62)). DEAR1 has been shown to be a novel tumor suppressor and polarity regulator [28, 29]. Loss of DEAR1 gene expression in breast cancer tissues is associated with a higher risk of recurrence in early -onset breast cancer [28]. DEAR1 has also been shown to regulate TGF-beta mediated epithelial mesenchymal transitionn (EMT) [29]. Kim *et al*. observed that, in several cell lines, the minor allele of rs12803915 significantly alters the cellular processing of pre-miR-612 and, consequently, the expression levels of mature miR-612 [30]. In addition, a recent study reported this SNP as significantly associated with acute lymphoblastic leukemia susceptibility [31]. Other recent studies found that miR-612 suppresses the invasive-metastatic cascade in hepatocellular carcinoma [32, 33]. However, so far, no reports indicate that miR-412 and miR-612 play a role in PC or the implicated SNPs alter the levels of these microRNAs. Further studies that measure these microRNA levels in PC tissues and investigate if the levels of these microRNAs correlate with these SNPs should be conducted to confirm our results.

Two of the significant SNPs are potentially functional as they are in the 3′-UTR. The SNP rs6128327 is located in the 3′-UTR region of *RAB22A* and may be of functional relevance because it is located in an exonic splice enhancer sequence as predicted by SNPinfo [34]. *RAB22A* gene expression was reported to be significantly increased in breast cancers compared with normal breast tissue. Elevated *RAB22A* mRNA levels in primary breast cancers were associated with significantly decreased overall survival and distant metastasis-free survival [35]. The SNP rs3217992 is located in the 3′-UTR region of *CDKN2B* and also in DNase I hypersensitivity site. It is predicted to affect mRNA stability and translation. Loss of *CDKN2B* is a very frequent event in several cancers, including PC [36–38].

The remaining SNPs that we identified are located in the intronic regions of genes. The ENCODE project has reported, after systematically mapping regions of transcription, transcription factor association, chromatin structure, and histone modification, that 80% of the genome is related to some biochemical function [39]. We found many intronic SNPs mapped to areas of histone modification, DNase I hypersensitivity, and altered transcription factor binding sites. Modification of histone proteins can influence gene expression by changing how accessible the chromatin is to transcription. Eighty percent of DNase hypersensitivity sites map to regions of genome expected to contain gene regulatory elements, including CpG islands and highly conserved sequences, and functions as promoter, silencer, insulator, *cis*- or *trans*-regulatory elements, or epigenetic signals [40]. Transcription factors play key roles in transcriptional regulation by controlling gene expression. We also did *cis*-eQTL analysis based on public data sets and found that rs4940086, rs7799635, rs1559849, rs6128327, and rs3217992 were significantly associated with gene expression. Together, these results point to potentially important functional regulatory variation.

There is currently no biomarker which can be accurately used to identify persons at risk of PC. When diagnosed early, it may be more possible to effectively treat, while most patients are diagnosed at later stages. Therefore, Biomarkers accurate enough to detect PC in the earliest stages are urgently needed. For individuals who are at increased risk due to genetic factors, effective early screening methods are especially important. Our study found a panel of SNPs associated with earlier onset of PC. Since the SNPs identified herein increase risk for early onset disease, it would be interesting for future studies to examine hereditary cancers that predispose to PC to determine whether these SNPs are associated with earlier ages of PC development or disease development itself since previous studies provided strong evidence of an increased risk of PC in BRCA2 mutation carriers [41, 42].

In conclusion, we analyzed functional genomic data sets to identify SNPs in the 3p12 pathway genes and genes related to the chromosome 20q interval and systematically assessed the associations of a large panel of tagging SNPs in the genes with age at diagnosis of PC. Compared with genome-wide association studies, a pathway-based approach improves the efficiency of identifying disease variants by jointly considering variants of the genes that belong to the same biological pathway. It reduces the number of false-positive findings and increases the effective power of the study by restricting analyses to SNPs in specific pathways and reduces the number of multiple tests. In addition, because the genes we selected from functional genomic datasets were frequently differentially/aberrantly expressed in PC, SNPs in these genes are more likely to be detected for associations with age at diagnosis of PC. Furthermore, we used two-phase study design. By adding the validated phase to the study design, our study is sufficiently powered to scrutinize false-positive findings. These findings require further replication and functional validation. The panel of SNPs identified may serve as susceptibility markers for earlier PC diagnosis, which is important for improving the prognosis of this disease. The findings of this study will contribute to our long-term goal to develop a risk model for PC and hopefully lead to early detection by allowing us to identify those individuals who will develop PC at an earlier age based on a risk score. If these findings are confirmed, these genetic variations may have utility as a panel of risk markers that could, combined with other genetic risk factors, be used as a screening tool to screen individuals who are more likely to develop PC at a younger age and recommended clinical surveillance. Such clinical application could lead to earlier detection and treatment, longer survival time, and lower mortality.

## METHODS

### Study cohort

We identified 1956 newly- diagnosed and histopathologically confirmed pancreatic adenocarcinoma patients, who were consecutively recruited at The University of Texas MD Anderson Cancer Center (Houston, TX) or the University of Pittsburgh (Pittsburgh, PA) from February 1999 to August 2004. To avoid heterogeneity attributable to racial differences in allele frequencies, the analysis was limited to 1729 self-reported non-Hispanic white individuals, 1279 from MD Anderson Cancer Center and 450 from the University of Pittsburgh. The study was approved by the Institutional Review Boards of both institutions, and all participants provided written informed consent for contributing blood for this research. DNA of patients was extracted with an AUTOPURE LS Automated DNA Purification Instrument (QIAGEN, Inc.) according to the manufacturer's instructions.

### Gene selection

We first analyzed a functional genomic dataset to identify genes from the targeted pathways involving 3p12 [11]. The 3p pathway dataset of genes differentially expressed in PC tumor versus normal samples and representing the 3p12 pathway to tumorigenesis have been described [5, 11, 26].

The 20q pathway genes were identified with integrated comparative genomic hybridization (CGH) and expression array analyses of PC cell lines and two primary tumor datasets (unpublished). Genes analyzed are in the copy number altered (CNA) genomic intervals with 2 fold or greater change in expression (*P* < 0.05). We also included microRNA (miRNA) genes predicted to be involved in the regulation of these pathways genes. miRNA prediction programs (microRNA.org, Targetscan, and Diana-microT v3.0) were used to identify miRNAs that are known or predicted to target the candidate biomarkers. We finally selected 135 genes that interact in the 3p12 pathway and pathways affected by altered gene expression in the 20q interval.

### SNP selection and genotyping

We used SNPbrowser version 4.0 (Life Technologies, Grand Island, NY) [43] to select tagging SNPs. This software was designed for selection of SNPs based on observed linkage disequilibrium (LD) through construction of metric LD maps and selection of haplotype tagging SNPs. The application provides easy and intuitive selection of SNPs, including visualization of SNPs, by showing gene structure, LD map, and haplotype block information. SNP selection was based on the ethnicity-specific LD patterns identified by the HapMap Project (http://hapmap.ncbi.nlm.nih.gov/). The tagging SNPs chosen had an r^2^ of 0.80 or more and a minor allele frequency (MAF) of 0.05 or more in the white population. SNPs from the adjacent 10-kb regions on either side of the gene were also included.

In the discovery phase (phase 1), Illumina's Golden Gate SNP genotyping assay (Illumina, San Diego, CA) was developed to examine SNPs that were assayable (design score > 0.60) according to the GoldenGate genotyping platform criteria. Genotypes were called using Beadstudio software (Illumina). Plates were constructed with duplicate and quality control samples. Twenty-four duplicated DNA samples were included for genotyping quality control. The average discordance rate of duplicates was 0.06%. We removed SNPs with an MAF of 0.01 or less, with a call rate < 95%, with discordance between duplicates, or with Hardy-Weinberg equilibrium with a *P*-value of 10^−5^ or less. A total of 1240 SNPs were included in the final analysis of association with age in phase 1. The SNPs that were significantly associated with age onset in phase 1 were further genotyped in phase 2 by using the Illumina BeadXpress platform according to the manufacturer's protocol. The BeadXpress system offers a cost effective platform for low- to mid- plex Golden Gate SNP genotyping assay using VeraCode technology.

### Statistical analysis

The outcome variable for the phase 1 and phase 2 as well as a pooled analysis was time to onset of PC. We used Cox proportional hazard regression analysis to test the association of each of the SNPs with age-associated risk of PC. All association analyses were adjusted for sex, history of smoking and alcohol use, presence of diabetes, and institutions, when appropriate. The Kaplan-Meier product-limit estimator was used to plot time to onset and the log-rank test to test for homogeneity of the survival curves by genotype for each of the SNPs. We examined the risk of each SNP by additive, dominant, and recessive models. The best-fitting model was the one with the smallest *P*-value among the three models. A combined analysis tested the underlying hypothesis that individuals with a larger number of unfavorable (risk-increasing) genotypes would be at higher risk for developing PC at a younger age. Unfavorable genotypes were defined on the basis of the genetic model that attained significance in the Cox regression. A Benjamini-Hochberg multiple testing correction threshold with false discovery rate of 10% was used to identify significant associations [44]. STATA software (version 10, StataCorp LP, College Station, TX) was used to perform the analyses.

### SNP function annotation

We explored the functional consequences of the SNPs using custom tracks on the UCSC Genome browser (http://genome.ucsc.edu) [45]. The UCSC Genome browser incorporates visualization of some of the Encyclopedia of DNA elements (ENCODE) functional elements, such as regions of transcription, transcription factor binding motifs, chromatin structure, CpG site methylation, and histone modification [39]. We also used online tools F-SNP [46], HaploReg [47], and RegulomeDB [48] to confirm each SNP in relation to annotated protein-coding genes and/or non-coding RNA genes. We investigated expression quantitative trait loci (eQTL) associations for the significant SNPs using Genevar, a database and Java tool designed for data analysis of SNP-gene associations in eQTL studies integrating multiple datasets [49].

## SUPPLEMENTARY MATERIALS FIGURE AND TABLES



## References

[1] Siegel RL, Miller KD, Jemal A (2016). Cancer statistics, 2016. CA Cancer J Clin.

[2] Howlader N NA, Krapcho M, Garshell J, Miller D, Altekruse SF, Kosary CL, Yu M, Ruhl J, Tatalovich Z, Mariotto A, Lewis DR, Chen HS, Feuer EJ (2015). SEER Cancer Statistics Review, 1975–2012.

[3] Shridhar R, Shridhar V, Wang X, Paradee W, Dugan M, Sarkar F, Wilke C, Glover TW, Vaitkevicius VK, Smith DI (1996). Frequent breakpoints in the 3p14. 2 fragile site, FRA3B, in pancreatic tumors. Cancer research.

[4] Sanchez Y, el-Naggar A, Pathak S, Killary AM (1994). A tumor suppressor locus within 3p14-p12 mediates rapid cell death of renal cell carcinoma *in vivo*. Proceedings of the National Academy of Sciences of the United States of America.

[5] Zhang K, Lott ST, Jin L, Killary AM (2007). Fine mapping of the NRC-1 tumor suppressor locus within chromosome 3p12. Biochemical and biophysical research communications.

[6] Lott ST, Lovell M, Naylor SL, Killary AM (1998). Physical and functional mapping of a tumor suppressor locus for renal cell carcinoma within chromosome 3p12. Cancer research.

[7] Lott ST, Chandler DS, Curley SA, Foster CJ, El-Naggar A, Frazier M, Strong LC, Lovell M, Killary AM (2002). High frequency loss of heterozygosity in von Hippel-Lindau (VHL)-associated and sporadic pancreatic islet cell tumors: evidence for a stepwise mechanism for malignant conversion in VHL tumorigenesis. Cancer Res.

[8] Tabach Y, Kogan-Sakin I, Buganim Y, Solomon H, Goldfinger N, Hovland R, Ke XS, Oyan AM, Kalland KH, Rotter V, Domany E (2011). Amplification of the 20q chromosomal arm occurs early in tumorigenic transformation and may initiate cancer. PloS one.

[9] Hoglund M, Gisselsson D, Hansen GB, Mitelman F (2004). Statistical dissection of cytogenetic patterns in lung cancer reveals multiple modes of karyotypic evolution independent of histological classification. Cancer genetics and cytogenetics.

[10] Kowalski J, Morsberger LA, Blackford A, Hawkins A, Yeo CJ, Hruban RH, Griffin CA (2007). Chromosomal abnormalities of adenocarcinoma of the pancreas: identifying early and late changes. Cancer genetics and cytogenetics.

[11] Balasenthil S, Chen N, Lott S, Chen J, Carter J, Grizzle WE, Frazier ML, Sen S, Killary AM (2011). A Migration Signature and Plasma Biomarker Panel for Pancreatic Adenocarcinoma. Cancer Prev Res.

[12] Liu Q, Chen J, Mai B, Amos C, Killary AM, Sen S, Wei C, Frazier ML (2012). A single-nucleotide polymorphism in tumor suppressor gene SEL1L as a predictive and prognostic marker for pancreatic ductal adenocarcinoma in Caucasians. Molecular carcinogenesis.

[13] Chen J, Li D, Wei C, Sen S, Killary AM, Amos CI, Evans DB, Abbruzzese JL, Frazier ML (2007). Aurora-A and p16 polymorphisms contribute to an earlier age at diagnosis of pancreatic cancer in Caucasians. Clinical cancer research.

[14] Chen J, Killary AM, Sen S, Amos CI, Evans DB, Abbruzzese JL, Frazier ML (2008). Polymorphisms of p21 and p27 jointly contribute to an earlier age at diagnosis of pancreatic cancer. Cancer letters.

[15] Dimas AS, Deutsch S, Stranger BE, Montgomery SB, Borel C, Attar-Cohen H, Ingle C, Beazley C, Gutierrez Arcelus M, Sekowska M, Gagnebin M, Nisbett J, Deloukas P (2009). Common regulatory variation impacts gene expression in a cell type-dependent manner. Science.

[16] Nica AC, Parts L, Glass D, Nisbet J, Barrett A, Sekowska M, Travers M, Potter S, Grundberg E, Small K, Hedman AK, Bataille V, Tzenova Bell J (2011). The architecture of gene regulatory variation across multiple human tissues: the MuTHER study. PLoS genetics.

[17] Stranger BE, Montgomery SB, Dimas AS, Parts L, Stegle O, Ingle CE, Sekowska M, Smith GD, Evans D, Gutierrez-Arcelus M, Price A, Raj T, Nisbett J (2012). Patterns of cis regulatory variation in diverse human populations. PLoS genetics.

[18] Liu Q, Chen J, Wang J, Amos C, Killary AM, Sen S, Wei C, Frazier ML (2014). Putative tumor suppressor gene SEL1L was downregulated by aberrantly upregulated hsa-mir-155 in human pancreatic ductal adenocarcinoma. Molecular carcinogenesis.

[19] Barberis MC, Roz E, Biunno I (2006). SEL1L expression in prostatic intraepithelial neoplasia and adenocarcinoma: an immunohistochemical study. Histopathology.

[20] Granelli P, Cattaneo M, Ferrero S, Bottiglieri L, Bosari S, Fichera G, Biunno I (2004). SEL1L and squamous cell carcinoma of the esophagus. Clinical cancer research.

[21] Cattaneo M, Orlandini S, Beghelli S, Moore PS, Sorio C, Bonora A, Bassi C, Talamini G, Zamboni G, Orlandi R, Menard S, Bernardi LR, Biunno I (2003). SEL1L expression in pancreatic adenocarcinoma parallels SMAD4 expression and delays tumor growth *in vitro* and *in vivo*. Oncogene.

[22] Cattaneo M, Fontanella E, Canton C, Delia D, Biunno I (2005). SEL1L affects human pancreatic cancer cell cycle and invasiveness through modulation of PTEN and genes related to cell-matrix interactions. Neoplasia.

[23] Orlandi R, Cattaneo M, Troglio F, Casalini P, Ronchini C, Menard S, Biunno I (2002). SEL1L expression decreases breast tumor cell aggressiveness *in vivo* and *in vitro*. Cancer research.

[24] Ferrero S, Falleni M, Cattaneo M, Malferrari G, Canton C, Biagiotti L, Maggioni M, Nosotti M, Coggi G, Bosari S, Biunno I (2006). SEL1L expression in non-small cell lung cancer. Human pathology.

[25] Duan R, Pak C, Jin P (2007). Single nucleotide polymorphism associated with mature miR-125a alters the processing of pri-miRNA. Human molecular genetics.

[26] Hu Y, Yu CY, Wang JL, Guan J, Chen HY, Fang JY (2014). MicroRNA sequence polymorphisms and the risk of different types of cancer. Scientific reports.

[27] Gao W, Shen H, Liu L, Xu J, Xu J, Shu Y (2011). MiR-21 overexpression in human primary squamous cell lung carcinoma is associated with poor patient prognosis. Journal of cancer research and clinical oncology.

[28] Lott ST, Chen N, Chandler DS, Yang Q, Wang L, Rodriguez M, Xie H, Balasenthil S, Buchholz TA, Sahin AA, Chaung K, Zhang B, Olufemi SE (2009). DEAR1 is a dominant regulator of acinar morphogenesis and an independent predictor of local recurrence-free survival in early-onset breast cancer. PLoS medicine.

[29] Chen NY, Balasenthil S, Reuther J, Frayna A, Wang Y, Chandler DS, Abruzzo LV, Rashid A, Rodriquez J, Lozano G, Cao Y, Lokken E, Chen J (2013). DEAR1 is a Chromosome 1p35 Tumor Suppressor and Master Regulator of TGFb-Driven Epithelial-Mesenchymal Transition. Cancer Disc.

[30] Kim HK, Prokunina-Olsson L, Chanock SJ (2012). Common genetic variants in miR-1206 (8q24.2) and miR-612 (11q13.3) affect biogenesis of mature miRNA forms. PloS one.

[31] Gutierrez-Camino A, Lopez-Lopez E, Martin-Guerrero I, Pinan MA, Garcia-Miguel P, Sanchez-Toledo J, Carbone Baneres A, Uriz J, Navajas A, Garcia-Orad A (2014). Noncoding RNA-related polymorphisms in pediatric acute lymphoblastic leukemia susceptibility. Pediatric research.

[32] Tang J, Tao ZH, Wen D, Wan JL, Liu DL, Zhang S, Cui JF, Sun HC, Wang L, Zhou J, Fan J, Wu WZ (2014). MiR-612 suppresses the stemness of liver cancer via Wnt/beta-catenin signaling. Biochemical and biophysical research communications.

[33] Tao ZH, Wan JL, Zeng LY, Xie L, Sun HC, Qin LX, Wang L, Zhou J, Ren ZG, Li YX, Fan J, Wu WZ (2013). miR-612 suppresses the invasive-metastatic cascade in hepatocellular carcinoma. The Journal of experimental medicine.

[34] Xu Z, Taylor JA (2009). SNPinfo: integrating GWAS and candidate gene information into functional SNP selection for genetic association studies. Nucleic acids research.

[35] Wang T, Gilkes DM, Takano N, Xiang L, Luo W, Bishop CJ, Chaturvedi P, Green JJ, Semenza GL (2014). Hypoxia-inducible factors and RAB22A mediate formation of microvesicles that stimulate breast cancer invasion and metastasis. Proceedings of the National Academy of Sciences of the United States of America.

[36] Lubomierski N, Kersting M, Bert T, Muench K, Wulbrand U, Schuermann M, Bartsch D, Simon B (2001). Tumor suppressor genes in the 9p21 gene cluster are selective targets of inactivation in neuroendocrine gastroenteropancreatic tumors. Cancer research.

[37] Kim WY, Sharpless NE (2006). The regulation of INK4/ARF in cancer and aging. Cell.

[38] Lindberg D, Akerstrom G, Westin G (2008). Evaluation of CDKN2C/p18, CDKN1B/p27 and CDKN2B/p15 mRNA expression, and CpG methylation status in sporadic and MEN1-associated pancreatic endocrine tumours. Clinical endocrinology.

[39] Consortium EP (2012). An integrated encyclopedia of DNA elements in the human genome. Nature.

[40] Crawford GE, Holt IE, Whittle J, Webb BD, Tai D, Davis S, Margulies EH, Chen Y, Bernat JA, Ginsburg D, Zhou D, Luo S, Vasicek TJ (2006). Genome-wide mapping of DNase hypersensitive sites using massively parallel signature sequencing (MPSS). Genome research.

[41] Moran A, O'Hara C, Khan S, Shack L, Woodward E, Maher ER, Lalloo F, Evans DG (2012). Risk of cancer other than breast or ovarian in individuals with BRCA1 and BRCA2 mutations. Familial cancer.

[42] Breast Cancer Linkage C (1999). Cancer risks in BRCA2 mutation carriers. Journal of the National Cancer Institute.

[43] De La Vega FM, Isaac HI, Scafe CR (2006). A tool for selecting SNPs for association studies based on observed linkage disequilibrium patterns. Pacific Symposium on Biocomputing Pacific Symposium on Biocomputing.

[44] Benjamini YH Y (1995). Controlling the False Discovery Rate: A Practical and Powerful Approach to Multiple Testing. Journal of the Royal Statistical Society Series B (Methodological).

[45] Rosenbloom KR, Armstrong J, Barber GP, Casper J, Clawson H, Diekhans M, Dreszer TR, Fujita PA, Guruvadoo L, Haeussler M, Harte RA, Heitner S, Hickey G (2015). The UCSC Genome Browser database: 2015 update. Nucleic acids research.

[46] Lee PH, Shatkay H (2009). An integrative scoring system for ranking SNPs by their potential deleterious effects. Bioinformatics.

[47] Ward LD, Kellis M (2012). HaploReg: a resource for exploring chromatin states, conservation, and regulatory motif alterations within sets of genetically linked variants. Nucleic acids research.

[48] Boyle AP, Hong EL, Hariharan M, Cheng Y, Schaub MA, Kasowski M, Karczewski KJ, Park J, Hitz BC, Weng S, Cherry JM, Snyder M (2012). Annotation of functional variation in personal genomes using RegulomeDB. Genome research.

[49] Yang TP, Beazley C, Montgomery SB, Dimas AS, Gutierrez-Arcelus M, Stranger BE, Deloukas P, Dermitzakis ET (2010). Genevar: a database and Java application for the analysis and visualization of SNP-gene associations in eQTL studies. Bioinformatics.

