# Supplementary material for: Identification of genetic variants predictive of early onset pancreatic cancer through a population science analysis of functional genomic datasets
Source: Oncotarget. 2016 Jul 29;7(35):56480–90. doi: 10.18632/oncotarget.10924 (PMC5302929; doi:10.18632/oncotarget.10924)
Supplement: Supplementary file 1 [file oncotarget-07-56480-s001.pdf]

Identification of genetic variants predictive of early onset pancreatic cancer through a population science analysis of functional genomic datasets

Supplementary Materials

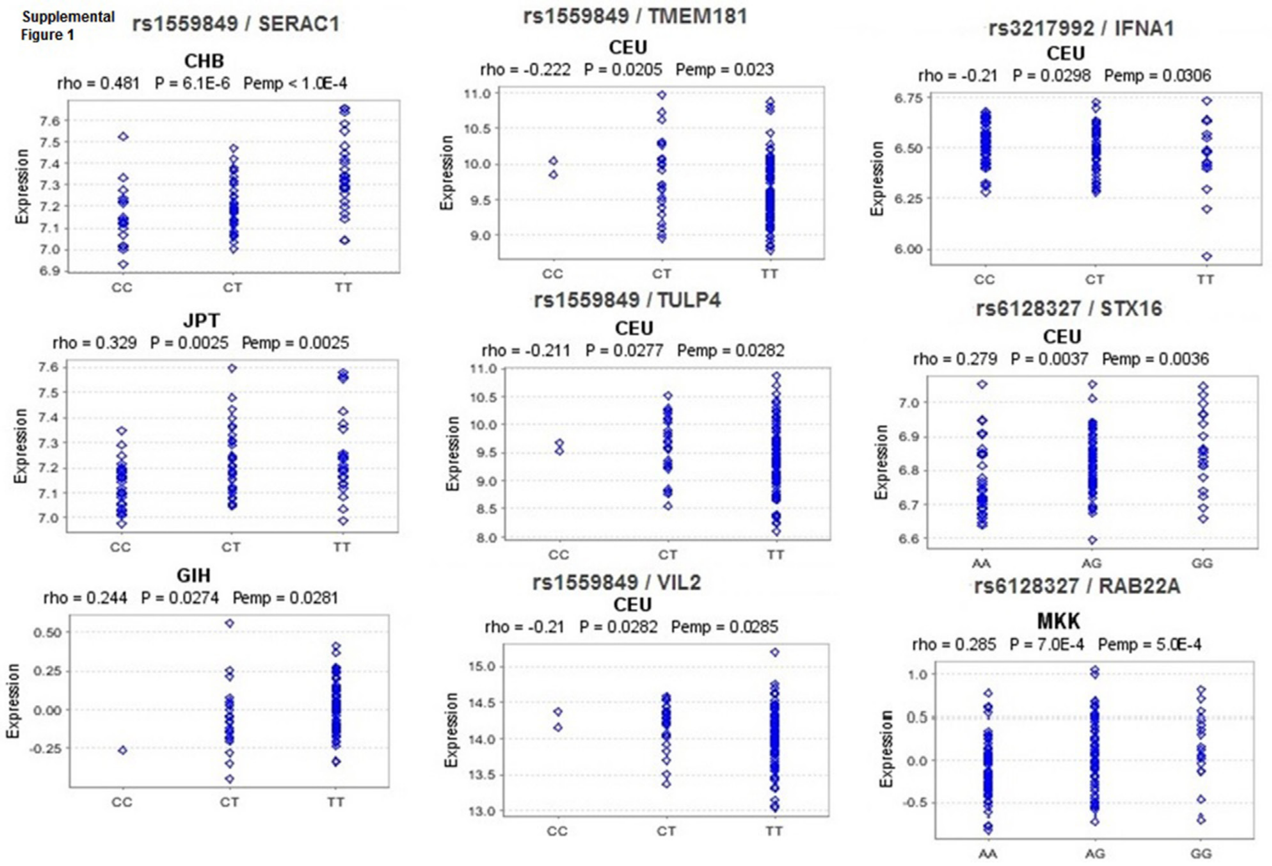

Supplementary Figure S1: eQTL analysis for SNPs and genes in the HapMap3 study. CEU: Utah residents with Northern and Western European ancestry from the CEPH collection; CHB: Han Chinese in Beijing, China; GIH: Gujarati Indians in Houston, Texas; JPT: Japanese in Tokyo, Japan; MKK: Maasai in Kinyawa, Kenya. Rho: Spearman correlation coefficient; *P*: *P*-values of Spearman correlation test; *P*emp: empirical *P*-values calculated from 10,000 permutations. The figures were downloaded from Genevar.

Supplementary Table S1: SNPs associated with age at diagnosis in discovery phase ($P < 0.05$)

Gere	SNP	*p* value Onset	Gere	SNP	*p* value Onset	Gere	SNP	*p* value Onset
ACSBG2	rsl6993465	0.0246	CDC42BPA	rs2104861	0.0115	HLA-DPB1	rs930	0.0369
ADAM32	rs6984223	0.0245	CDC42BPA	rs4653475	0.0156	IFI30	rs2921	0.0115
ADAM32	rsl2542595	0.0472	CDC42BPA	rs4653474	0.0297	IFNAR1	rs2298677	0.0176
AKAP12	rs 1042167	0.0086	CDKN1A	rs762623	0.0404	IFNAR1	rs2252930	0.0266
AN03	rs172435 66	0.0032	CDKN1A	rs3829963	0.0434	KDELR2	rs7799635	0.0416
ANTXR1	rs4S54547	0.0002	CDKN1B	rs309373 6	0.0273	KHDRBS1	rs3738002	0.0138
ANTXR1	rsl2713671	0.0053	CDKN1B	rs34329	0.0485	MCM7	rsl527423	0.0422
ANTXR1	rs12479401	0.0209	CDKN2B	rs3217992	0.0477	MIB1	rs9989532	0.0482
ANTXR1	rsll689690	0.0357	CMIP	rsll28658	0.0178	MIRN100I |TRNAK-UUU	rsl834306	0.0432
ARHGEF7	rs 3783083	0.0231	CMIP	rs9936936	0.0182	MIRN145	rs353291	0.0377
ARHGEF7	rs6692	0.0332	CMIP	rsl2447549	0.0255	MIRN29A	rs24168	0.0099
ARL6IPE	rs7039	0.0365	CMIP	rs16955460	0.0459	MIRN31| |L0C402359	rsl869205	0.0212
ATP6V1G1	rs 7868842	0.0135	CMIP	rs4889359	0.0494	MIRN331| |LOC643531	rslll07973	0.0497
ATPIF1	rs9508	0.0147	CTDSPL	rs7372209	0.0460	MIRN412	rs61992671	0.0155
B4GALT5	rs951497	0.0108	EFNA1	rsl2904	0.0361	MIRN612	rsl2803915	0.0387
B4GALT5	rs 6063425	0.0495	EPS&	rs4283039	0.0458	MIRNLET7A2| IMIRN100	rs543412	0.0293
BCAS1	rsl055253	0.0020	FAM62B/ESYT2	rs3816460	0.0302	MTAP	rs7047648	0.0133
BCAS1	rs2299721	0.0148	FAM62B/ESYT2	rsll53999	0.0485	MTAP	rs4478653	0.0275
BCAS1	rs208365	0.0158	FBN2	rs7288	0.0473	NATIO	rs12419404	0.0220
BCAS1	rs 81484	0.0242	FU11806/Z C3 H14	rs2116445	0.0003	NEIL1	rs5745925	0.0468
BCAS1	rs208389	0.0301	FU11806/Z C3 H14	rs2297124	0.0118	NRP1	rs734186	0.0317
BCAS1	rs458990	0.0392	FU11806/Z C3 H14	rs4904460	0.0182	NRP1	rsl555319	0.0371
BCAS1	rs 6013871	0.0460	FU11806/Z C3 H14	rs10144475	0.0278	NRP2	rs6711044	0.0276
BCAS1	rs 6097745	0.0476	FU31818| |C7orf60	rs7779708	0.0170	NRP2	rs3771047	0.0404
C7orf60	rs17484145	0.0171	FU31818| |C7orf60	rs821785	0.0224	NRP2	rs3771044	0.0404
NRP2	rs10773	0.0431	SMAD3	rs4147358	0.0377	TGFBR2	rs304839	0.0311
NRP2	rs 849555	0.0452	SMAD3	rsll071937	0.0419	TGFBR2	rs9310940	0.0348
PAPPA	rs 3 810940	0.0327	SMAD3	rsl6950687	0.0439	TGFBR2	rs4955212	0.0410
PIP5K3	rs4673402	0.0050	SMAD3	rs9972423	0.0473	TGFBR2	rs3773 644	0.0441
PIP5K31 |IDH1	rs 10207062	0.0322	SMAD3	rsl5974	0.0258	TGFBR2	rsll924422	0.0486
PIWIL1	rs1106042	0.0333	SMAD4	rs2282544	0.0071	TNC	rsl537015	0.0330
PPARGC1B	rs1076064	0.0107	SMAD7	rs3764482	0.0398	TNRC6B	rs9623117	0.0391
PSMD2	rs6845	0.0021	SOD1/SCAF4	rs202449	0.0309	TP53	rsl2951053	0.0321
PTP4A2	rsl2739839	0.0007	STAT3	rs3809 75 8	0.0062	TPST2	rs4275	0.0337
PTPRO	rsll056575	0.0241	SYNGR2/TK1	rs2854702	0.0064	TUBA11 |STK16	rs8447	0.0411
RAB22A	rs6128327	0.0077	SYNGR2/TK1	rsl065769	0.0198	UBQLN3	rs10605 74	0.0330
RAB23	rsl2195875	0.0076	TAOK1	rs508706	0.0267	VEGFA	rsl0434	0.0386
RAB23	rsl2211611	0.0215	TFPI	rs3213739	0.0030	VEGFA	rs21463 2 3	0.0443
RAB23	rs 9 3 57940	0.0421	TFPI	rsll674362	0.0119	WWTR1	rs4681524	0.0033
RAN	rsl4035	0.0248	TFPI	rs8176508	0.0145	WWTR1	rs7616772	0.0081
RHOF	rs7308123	0.0395	TFPI	rs2041778	0.0322	WWTR1	rs98583 54	0.0125
SCRN1	rs 69 76789	0.0458	TFPI	rs8176605	0.0350	WWTR1	rs6806548	0.0224
SEL1L	rsl2436488	0.0332	TFPI	rsl2613071	0.0355	WWTR1	rs177879 5 3	0.0266
SERAC1	rsll756587	0.0315	TFPI	rs2192824	0.0355	WWTR1	rs9820948	0.0370
SERAC1	rsl559849	0.0381	TFPI	rsl2693471	0.0388	WWTR1	rs982873 2	0.0409
SERAC1	rs890458	0.0444	TFPI	rsl3427829	0.0428	WWTR1	rs9861157	0.0434
SMAD2	rs4940086	0.0397	TFPI	rsl3035938	0.0494	XPC	rs2228001	0.0309
SMAD2	rsl792689	0.0459	TGFBR2	rsl346907	0.0028	ZNF217	rs2766669	0.0048
SMAD3	rs745103	0.0373	TGFBR2	rs3773663	0.0118	ZNF217	rs6063966	0.0114
SMAD3	rsll637659	0.0374	TGFBR2	rs2276768	0.0214			

Supplementary Table S2: SNPs significantly associated with age at pancreatic cancer diagnosis validated in phase 2

Gene	SNP ID	Minor Allele	No. of Genotype[a]	Genetic Model	Adjusted HR[b](95% CI)	*P*
miR-412	rs61992671	G	363/613/273	Dominant	1.28 (1.14–1.45)	**7.19E-05**
SERAC1	rs1559849	G	945/277/27	Recessive	1.85 (1.26–2.72)	0.001
SMAD3	rs11071937	A	570/559/120	Recessive	1.29 (1.06–1.55)	0.009
KDELR2	rs7799635	G	791/395/63	Additive	0.89 (0.81–0.98)	0.013
ZNF217	rs2766669	G	903/326/20	Dominant	1.16 (1.02–1.31)	0.021
RAB22A	rs6128327	G	453/583/213	Additive	1.10 (1.01–1.22)	0.023
CDKN2B	rs3217992	A	462/587/200	Recessive	0.84 (0.72–0.98)	0.028
ARHGEF7	rs6692	A	898/316/35	Recessive	0.69 (0.48–0.98)	0.036
miR-612	rs12803915	A	754/434/59	Additive	1.11 (1.01–1.22)	0.038
miR-145	rs353291	G	466/584/199	Additive	1.09 (1.01–1.18)	0.039
SMAD2	rs4940086	G	602/536/111	Additive	1.10 (1.00–1.20)	0.040

[a]The number of participants for the genotype: WW (wild-type)/WM (heterozygous)/MM (homozygous variant).
[b]Adjusted by sex, smoking status, alcohol status, and diabetes status.
The significant results after multiple test correction (false discovery rate *q*-value < 0.1) are shown in bold.
SNP, single nucleotide polymorphism; HR, hazard ratio; CI, confidence interval.

Supplementary Table S3: SNPs significantly associated with age at diagnosis of pancreatic cancer in the pooled analysis

Gene	SNP ID	Minor Allele	No. of Genotype[a]	Genetic Model	Adjusted HR[b](95% CI)	*P*
miR-412	rs61992671	G	505/837/387	Dominant	1.29 (1.16–1.43)	**2.09E–06**
ZNF217	rs2766669	G	1244/453/32	Dominant	1.20 (1.08–1.33)	**8.11E–04**
RAB22A	rs6128327	G	633/800/294	Additive	1.12 (1.05–1.20)	**0.001**
SMAD4	rs2282544	C	1525/192/12	Additive	1.23 (1.08–1.41)	**0.003**
PPARGC1B	rs1076064	G	637/807/282	Recessive	1.22 (1.07–1.38)	**0.003**
KDELR2	rs7799635	G	1086/562/81	Additive	0.89 (0.82–0.96)	**0.003**
SMAD2	rs4940086	G	802/760/167	Additive	1.11 (1.04–1.20)	**0.003**
CDKN2B	rs3217992	A	633/816/279	Recessive	0.83 (0.73–.95)	**0.005**
NRP2	rs849555	G	720/799/210	Recessive	1.22 (1.05–1.41)	0.008
STK16	rs8447	G	801/764/163	Dominant	1.13 (1.03–1.25)	0.009
miR-612	rs12803915	A	1047/604/76	Additive	1.11 (1.02–1.21)	0.012
CDC42BPA	rs4653475	A	918/684/126	Additive	0.91 (0.84–0.98)	0.015
BCAS1	rs1055253	G	553/870/306	Dominant	0.88 (0.80–0.98)	0.015
ANTXR1	rs11689690	A	788/750/188	Additive	1.09 (1.02–1.17)	0.017
SERAC1	rs1559849	G	1303/389/37	Recessive	1.47 (1.06–2.04)	0.021
PAPPA	rs3810940	G	863/703/163	Recessive	1.21 (1.02–1.42)	0.022
WWTR1	rs4681524	G	645/827/257	Recessive	1.16 (1.02–1.33)	0.024
BCAS1	rs6097745	A	985/648/96	Additive	1.09 (1.01–1.18)	0.029
ZC3H14	rs10144475	G	501/834/393	Recessive	1.13 (1.01–1.27)	0.029
CMIP	rs1128658	A	1409/295/24	Recessive	0.64 (0.43–0.96)	0.030
ANTXR1	rs4854547	G	699/782/248	Recessive	0.87 (0.76–0.99)	0.035
EFNA1	rs12904	A	622/829/277	Recessive	1.15 (1.01–1.31)	0.036
RAB23	rs12211611	C	787/409/53	Recessive	1.26 (1.01–1.58)	0.042
ZC3H14	rs2116445	A	862/347/40	Recessive	0.76 (0.58–1.00)	0.046
CDC42BPA	rs4653474	G	746/441/62	Recessive	0.80 (0.65–1.00)	0.046

[a]The number of participants for the genotype: WW (wild-type)/WM (heterozygous)/MM (homozygous variant).
[b]Adjusted by sex, smoking status, alcohol status, diabetes status, and study phase.
The significant results after multiple test correction (false discovery rate *q*-value < 0.1) are in bold.
SNP, single nucleotide polymorphism; HR, hazard ratio; CI, confidence interval.
